# Chronic wound isolates and their minimum inhibitory concentrations against third generation cephalosporins at a tertiary hospital in Uganda

**DOI:** 10.1038/s41598-021-04722-6

**Published:** 2022-01-24

**Authors:** Khalim Wangoye, James Mwesigye, Martin Tungotyo, Silvano Twinomujuni Samba

**Affiliations:** 1 Department of Pharmacy and Pharmacology, Mbarara University of Science and Technology, P.O Box 1410, Mbarara, Uganda; 2 Department of Medical Laboratory Science, Mbarara University of Science and Technology, P.O Box 1410, Mbarara, Uganda; 3 Department of Surgery, Mbarara University of Science and Technology, P.O Box 1410, Mbarara, Uganda

**Keywords:** Microbiology, Health care, Medical research

## Abstract

Globally, the burden of chronic wound infections is likely to increase due to the rising levels of bacterial resistance to antibiotics. In the United States of America alone, more than 6.5 million chronic wounds with evidence of bacterial infection are diagnosed every year. In addition, the polymicrobial environment in chronic wound infections has been observed from several studies as a risk factor for development of resistance to many antibiotics including the third generation cephalosporins currently used in Mbarara Regional Referral Hospital for treatment of chronic wound infections. Therefore the main objective of this study was to determine the prevalence of chronic wound isolates and their minimum inhibitory concentrations (MIC) against third generation cephalosporins. This study was a cross-sectional descriptive and analytical survey of bacterial isolates from chronic wound infection among 75 study participants admitted in the surgical ward of Mbarara Regional Referral Hospital (MRRH), a tertiary Hospital in Western Uganda. Standard laboratory bacterial culture and identification techniques as well as broth microdilution method were used to isolate, identify pathogens and test for MIC respectively. We found tha**t** 69/75 study participants had samples with bacterial growth and the most prevalent pathogens isolated were *staphylococcus aureus* (40.6%) *and Klebsiella* spp. (29%). Generally, most isolates were susceptible to cefoperazone + sulbactum 2 g (Sulcef) and ceftriaxone 1 g (Epicephin). The overall prevalence of isolates in chronic wound infection among patients admitted in the surgical ward of MRRH was 92% and the most prevalent isolates were *Staphylococcus aureus, Klebsiella species* and *proteus species* respectively. The observed MIC values were higher than the CLSI clinical breakpoint, implying a decreasing trend in susceptibility of chronic wound isolates to third generation cephalosporins.

## Introduction

Globally, the burden of chronic wound infections is likely to increase due to the rising levels of bacterial resistance to antibiotics^1^. In the United States of America alone, more than 6.5 million chronic wounds with evidence of bacterial infection are diagnosed every year^2^. In particular, the risk of antibiotic resistance has been estimated to high in chronic wound infections because of the polymicrobial environment associated with chronic wound infections which creates favorable environment for exchange of resistance genes between microorganisms^3^.

Although third generation cephalosporins have been recommended as the drug of choice in the management of severe bacterial infections including chronic wound infections in Uganda’s clinical guidelines, 81% of the prescriptions reviewed indicates inappropriate use of ceftriaxone (particularly wrong diagnosis), increasing the risk of bacterial resistance to ceftriaxone and other third generation cephalosporins^4^.

Moreover, routine culture and sensitivity tests and periodic antibiotic resistance surveillance studies are rarely performed in Mbarara Regional Referral Hospital due to inadequate microbiology supplies and the turnaround time for culture and sensitivity tests is high in the majority of Hospitals in Uganda, causing delays in making clinical decisions required for selection of effective third generation cephalosporins^5^.

Consequently, patients with chronic wound infections are likely to experience long hospital stays, high treatment costs, further delay of wound healing, development of severe invasive bacterial infections and increased emergency of antibiotics resistance if ineffective third generation cephalosprins are used.

Therefore this study was conducted to determine the prevalence of pathogens in chronic wound infections and their minimum inhibitory concentrations (MIC) against third generation cephalosporins, so as to guide Clinicians to make evidence-based prescription of third generation cephalosporins required for timely and effective management of chronic wound infections as well as dose optimization and individualization.

## Methods

### Study design

The study was a cross-sectional descriptive and analytical survey of bacterial isolates from chronic wound infections at the surgical ward of MRRH from August 2020 to October 2020.

### Study setting

Participants were enrolled from the surgical ward of MRRH between August 2020 and October 2020. MRRH is a public and teaching Hospital with a bed Capacity of 300 beds and it is a Regional Referral Hospital in Western Uganda located in Mbarara City, approximately 250 km from Kampala, the capital City of Uganda. Its catchment population is approximately 10 million people. The annual prevalence of chronic wound infection is approximately 420 patients. The surgical ward is currently run by 44 medical staff.

The microbiological procedures were carried out in the Microbiology Laboratory of Mbarara University of Science and Technology, Mbarara City, Uganda. The Microbiology Laboratory is well equipped and managed by three highly experienced staff that is 2 Laboratory technologists and 1 senior laboratory technologist. This laboratory is certified by the central public health laboratory of Uganda to perform a wide range of tests including culture and sensitivity.

### Study population

The study population was patients with chronic wound infections admitted at the Surgical Ward of MRRH in Uganda.

### Selection criteria

All inpatients admitted in the surgical ward with signs and symptoms of chronic wound infections (increasing pain at the ulcer site, erythema, edema, heat, purulent exudate, serous exudate, delayed ulcer healing, discolored granulation tissue, friable granulation tissue, wound base pocketing, foul odor and wound breakdown) and consented to participate in the study were included in this study while patients who expressed voluntary withdrawal during the course of this study were excluded.

### Sample size

The following assumptions were made during sample size calculation;

- Research data was collected for 3 months and the expected population of patients with chronic wound infection was 105 patients (approximately 35 patients per month) in accordance with the MRRH patient records of 2018.
- The prevalence of chronic wound infections was estimated to be 22%^6^.
  $${{\text{N}}_o} = {{\text{Z}}^2} * {\text{P}}\left( {1 - {\text{P}}} \right)/{{\text{E}}^2}$$

N_o_ = Sample size; Z = Confidence level; P = Estimated proportion of chronic wound infections in the population; E = Desired level of precision; Z = 1.96, P = 0.22 (22%), E = 0.05

$$\begin{aligned} {{\text{N}}_o} = \,& {1.96^2} * 0.22 \, \left( {1 - 0.22} \right)/{0.05^2} = 3.842 * 0.22 * 0.78/0.0025. \\ = \,& 264\;{\text{Patients}}. \\ \end{aligned}$$

Finite population correction^7^:

$${\text{n}} = {{\text{N}}_o}/\left[ {\left( {{N_o} - 1} \right)/{\text{N}}} \right] + 1,$$

n = Adjusted Sample size; N = Population size (105 patients);

$${\text{n}} = 264/\left[ {\left( {264 - 1} \right)/105} \right] + 1,\;\;\;n = 264/3.5 = 75\;{\text{patients}}.$$

### Sampling technique

Convenience sampling technique was used to select the study subjects who met the criteria for chronic wound infections^8^.

### Study procedures

For diagnosis of chronic wound infections, signs of chronic wound infection described under the selection criteria were used by the Clinician to guide the clinical diagnosis of chronic wound infection.

### Sample collection and bacterial identification

Two nurses working in the surgical ward were trained by an experienced laboratory technologist to empower them with skills of obtaining wound swabs for culture and sensitivity.

After obtaining an informed consent from the patients meeting the criteria, routine clinical samples were aseptically collected by a trained nurse from the patients’ wound base using sterile cotton swabs. The standard operating procedure developed by British Columbia Provincial Nursing Skin & Wound Committee were used to ensure an aseptic procedure^9^.The samples were transported to the Microbiology Laboratory of MUST within 30 min. Only one swab was obtained from each patient after cleaning the wound base with sterile normal saline.

### Laboratory procedures

I. Primary cultures: On receipt, swab specimens were registered in the laboratory research register.
II. Wound swabs were inoculated on chocolate agar, blood agar and MacConkey Agar as follows;
III. Using inoculating loop, each sample was streaked onto the upper one fourth portion of an agar plate with parallel overlapping strokes. The plates were labeled.
IV. The loop was flamed and allowed to cool. The plate was turned at right angle. Overlapped the previous streak once or twice and repeated the streaking process on one-half of the remaining area.
V. Procedure 4 was repeated.
VI. The plates were incubated overnight at 35 °C–37 °C in the incubator.
VII. After incubation for 16–20 h, the plates were checked for bacterial growth.
VIII. Representative bacterial colonies were selected based on the difference in shape, size and color. Selected colonies from each plate were sub-cultured and incubated overnight.
IX. Bacterial identification: This was performed based on morphological, cultural characteristics such as hemolysis on blood agar, swarming (positive for *proteus* spp.), changes in physical appearance on differential agar (pink appearance of lactose-fermenting bacterial colonies on macConkey agar), motility test was positive for *enterobacter agglomerans* and *providencia* spp. In addition, Table 1 shows the biochemical tests that were performed to confirm the identity of bacterial pathogens;

**Table 1:** Biochemical tests for Identification of bacterial pathogens.

Isolate	Biochemical test	Expected results
*Staphylococcus aureus*	Catalase	Positive
	Coagulase	Positive
	Mannitol fermentation	Positive
	Dnase	Positive
*Klebsiella* spp.	Citrate	Positive
	Urea	Positive
	Indole	Negative
*Proteus* spp.	Hydrogen sulphide	Positive
	Urea	Positive
	Citrate	Positive
	Oxidation	Positive
*Enterobacter agglomerans*	Hydrogen sulphide	Negative
	Urea	Negative
	Indole	Negative
*Providential* spp.	Indole, methyl red, citrate, nitrate reductase and catalase	Postive

### Antibacterial susceptibility testing

The minimum inhibitory concentrations and antibacterial susceptibility testing were performed using broth microdilution technique as described by CLSI and the review in the general principle and practices of antimicrobial susceptibility testing^10^. The Procedure for Broth microdilution involved the following steps;

I. Preparation of stock solutions: Stock solutions were prepared based on the manufacturer’s instruction for reconstitution. All the 5 antibiotic brands did not have potency information and the weight for antibiotics were calculated based on the highest plasma concentrations derived from the following pharmacokinetic studies because of the correlation that exist between MIC and pharmacokinetic parameters^11^. Table 2 shows the weight of antibiotics as calculated based on their respective maximum plasma concentrations.

**Table 2:** Weight of powder for stock solutions.

S/no	Antibiotic	Maximum plasma concentration (desired concentration) µg/ml	References	Weight of powder(g) (desired concentration) × volume of diluent(1000 ml) divide by 1,000,000 (g)
1	Ceftriaxone 1 g (Epicephin)	168	^12^	0.168
2	Cefoperazone + Sulbactam 2 g (Sulcef)	159	^13^	0.159
3	Cefotaxime 1 g (Omnatax)	41.1	^14^	0.0411
4	Cefpodoxime 200 mg (Ximeprox)	2.7	^15^	0.0027
5	Cefixime 400 mg (Gramocef-o 400)	2.47	^16^	0.00247

The antibiotic solutions were kept in the refrigerator at a temperature of 4 °C.

I. Using a pipette, 100 µl of sterile brain heart infusion were dispensed into the wells of microtitre plates, each row labeled to corresponding antibiotic.
II. 100 µl of the antibiotic stock solution were also dispensed into the well in column 1. Using the pipette set at 100 µl, mix the antibiotics into the wells in column 1 by sucking up and down 6 times.
III. 100 µl of this were withdrawn from column1 and added to column 2, making column 2 a two-fold dilution of column 1.
IV. 100 µl of column 2 were transferred to column 3. This was repeated down to column 9.
V. 5 µl of isolates suspended in sterile water and adjusted to McFarland turbidity (10^4^ × 10^5^ CFU/ml) were dispensed into the wells except wells in column 11 for sterility control. Wells in column 10 were used for growth control and contained 100 µl of brain heart infusion and 5 µl of isolates.
VI. Microtitre plates were then covered with sterile aluminum foil to prevent evaporation during incubation.
VII. After 24 h incubation at 37 °C, the microtitre plates were observed using a reading mirror for visible bacterial growth as indicated by turbidity. The smallest concentrations of antibiotics that inhibited growth were recorded as the MIC.

### Quality control

To ensure consistent and high quality research outputs, the researcher implemented quality control measures throughout the entire research process. Antibiotics for the third generation cephalosporins, culture media and staining reagents were procured from premises licensed by the National Drug Authority of Uganda to avoid the risk of counterfeit products which could affect the quality of research results. In- addition, the procured antibiotics, culture media and staining reagents were strictly stored at conditions specified by the manufacturers to avoid product deterioration during the research process.

All batches of culture media were tested for sterility and ability to support bacterial growth (using standard isolates; Escherichia *coli* American type culture collection (ATCC) 25,922 and *staphylococcus aureus* ATCC 25,923). Clinical breakpoints published by CLSI 2018 were used to interpret the observed MIC values and all microbiology procedures were conducted by registered laboratory technologists with 10 years of experience in microbiology laboratory work.

### Data processing and analysis plan

The study data was entered into Microsoft Excel and exported to STATA version 15.0 for statistical analysis. Frequencies, and mean (SD; standard deviation) were computed to summarize the data.

#### Objective 1

The prevalence of pathogens was first summarized as proportions and to determine the existence of any association between age group, gender of study participants and prevalence of specific pathogens, a Chi-Square/Fisher’s Exact test was computed. Results were presented in a bar graph and table.

#### Objective 2

The MIC values were summarized as mean ± SD and analyzed using one-way ANOVA (Analysis of Variance) to determine if there were significant differences between the mean MICs for the isolates with respect to the different groups of antibiotics studied and the final results were presented in a table. The level of significance was preset at 5% and *p*-values less than 0.05 were considered statistically significant in each of the above statistical tests.

### Ethical approval

This study was approved by the research ethics committee of Mbarara University of science and technology (Protocol registration number: 06/12-19). In addition, all methods were performed in accordance with the relevant guidelines/regulations and informed consent was obtained from all participants or legal guardians. To ensure privacy and safety of data, all information collected from study participants was stored under key and lock and electronic documents were pass word-protected.

## Results

### Overall prevalence of pathogens in study participants’ samples

**Figure 1:**
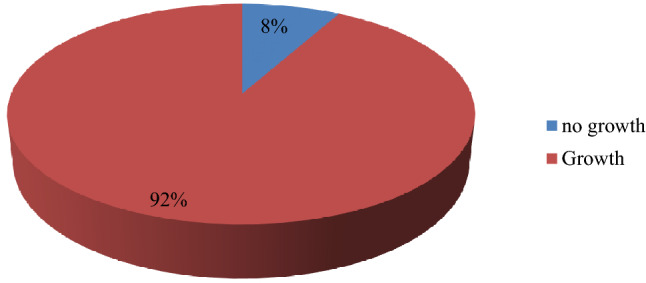
Overall prevalence of chronic wound infection isolates. This figure shows the overall prevalence of isolates in chronic wound infection. 75 wound swabs were inoculated on routine laboratory culture media for growth and pathogens were identified based on morphological, cultural characteristics and biochemical tests. Overall, 69 (92%) isolates were identified from 75 samples while 6 (8%) samples exhibited no growth.

A total of 75 samples were collected from 75 participants. The overall prevalence of pathogens was 69/75 (92%) while 6/75 (8%) of participants’ samples exhibited no growth (Fig. 1).

### Specific prevalence of pathogens isolated from chronic wound infections

**Figure 2:**
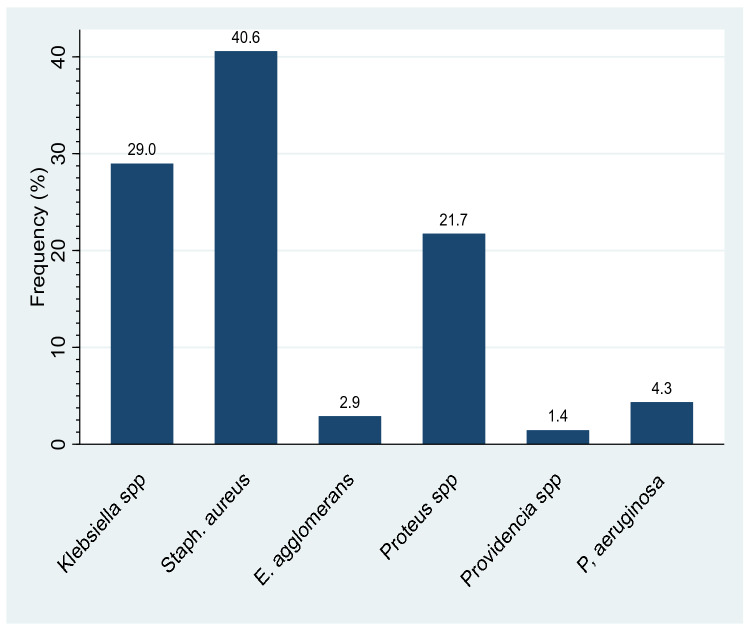
Specific prevalence of chronic wound infection isolates. This figure shows the specific prevalence of isolates in chronic wound infection. Seventy five (75) wound swabs were inoculated on routine laboratory culture media for growth and pathogens were identified based on morphological, cultural characteristics and biochemical tests. Six (6) types of isolates were identified in the following order of prevalence; *Staphylococcus aureus* (40.6%), *Klebsiella species* (29.0%), *Proteus species* (21.7%), *Pseudomonas aeruginosa* (4.3%), *Enterobacter agglomerans* (2.9%) and *Providentia species* (1.4%).

The data shows that of the 69 isolates recovered from 75 samples, the most prevalent pathogens isolated were *Staphylococcus aureus* (40.6%, n = 28/69) *Klebsiella* spp. (29%, n = 20/69) while the least prevalent pathogens included *Providencia* spp. (1.4%, n = 1/69) and *Enterobacter agglomerans* (2.9%, n = 2/69) see Fig. 2.

### Prevalence of bacterial isolates by age and gender of study participants

Table 3 shows the prevalence of pathogens that were isolated from the participants’ chronic wound infections with respect to age group and gender. Chronic wound infections caused by *staphylococcus aureus* were more prevalent among female patients (53.6%) aged below 40 years (67.9%) while chronic wound infections by *Klebsiella* spp. were most prevalent among male patients (60%) aged above 40 years(70%). However the prevalence of each bacterial isolate was not significantly associated with a particular age group or gender of the study participants (p-values > 0.05).

**Table 3:** Prevalence of pathogens isolated from chronic wound infections according to gender and age group of study participants.

Isolates (N = 69)	Frequency, n (%)		*P* value	Frequency, n (%)		*P* value
	≤ 40 yrs	> 40 yrs		Female	Male	
*Klebsiella species*	8 (40.0)	12 (60.0)	0.077	6 (30.0)	14 (70.0)	0.111
*Staphylococcus aureus*	19 (67.9)	9 (32.1)	0.116	15 (53.6)	13 (46.4)	0.233
*Enterobacter agglomerans*	1 (50.0)	1 (50.0)	0.850	1 (50.0)	1 (50.0)	0.884
*Proteus species*	8 (53.3)	7 (46.7)	0.778	7 (46.7)	8 (53.3)	0.878
*Providencia species*	1 (100.0)	0 (0.0)	0.565	1 (100.0)	0 (0.0)	0.449
*Pseudomonas aeruginosa*	2 (66.7)	1 (33.3)	0.599	1 (33.3)	2 (66.7)	0.577

yrs: years, n:number.

### The MICs of bacterial isolates to third generation cephalosporins

In Table 4, the mean differences in minimum inhibitory concentration of the pathogens with respect to the different brands of cephalosporin were tested using analysis of variance (ANOVA) or the F-test and the results are presented.

**Table 4:** The MICs for susceptible isolates to third generation cephalosporins.

Third generation cephalosporin	Bacterial isolate	Mean ± SD (µg/ml)	*P* Value
Ceftriaxone 1 g (Epicephin)	*Klebsiella species*	22.97 ± 27.15	0.2469
	*Staphylococcus aureus*	15.77 ± 23.32	
	*Enterobacter agglomerans*	47.25 ± 51.97	
	*Proteus species*	31.50 ± 14.85	
	*Pseudomonas aeruginosa*	44.65 ± 55.65	
Cefoperazone + Sulbactam 2 g (Sulcef)	*Klebsiella species*	2.91 ± 2.93	0.4144
	*Staphylococcus aureus*	4.23 ± 8.41	
	*Enterobacter agglomerans*	11.19 ± 12.32	
	*Proteus species*	8.60 ± 13.59	
	*Providencia species*	4.97 ± 0.00	
	*Pseudomonas aeruginosa*	8.50 ± 10.11	
Cefotaxime 1 g (Omnatax)	*Klebsiella species*	1.60 ± 1.71	0.1946
	*Staphylococcus aureus*	2.96 ± 4.66	
	*Enterobacter agglomerans*	0.72 ± 0.79	
	*Proteus species*	6.34 ± 7.97	
Cefixime 400 mg (Gramocef-0–400)	*Klebsiella species*	0.38 ± 0.48	0.8680
	*Staphylococcus aureus*	0.40 ± 0.53	
	*Enterobacter agglomerans*	0.04 ± 0.00	
	*Proteus species*	0.64 ± 0.00	
Cefpodoxime 200 mg (Ximeprox)	*Klebsiella species*	0.34 ± 0.47	0.6505
	*Enterobacter agglomerans*	0.70 ± 0.00	

In the ceftriaxone brand (Epicephin), *staphylococcus aureus* exhibited the lowest mean MIC (15.77 µg/ml) while a highest mean inhibitory concentration was observed for *Enterobacter agglomerans species* (47.25 µg/ml) followed by *pseudomonas aeruginosa* at 44.65.0 µg/ml. For the Cefoperazone + Sulbactam 2 g (Sulcef) brand, *Klebsiella species* had the lowest mean MIC (2.91 µg/ml) and the highest mean inhibitory concentration was exhibited by *Enterobacter agglomeran* (11.19 µg/ml)*, Proteus species* (8.60 µg/ml) and *Pseudomonas aeruginosa* (8.50 µg/ml) respectively. For Cefotaxime 1 g (Omnatax), the highest mean inhibitory concentration was for *Proteus specie* (6.3 µg/ml).

*Klebsiella species* (MIC = 0.34 µg/ml) and *Enterobacter agglomerans* (MIC = 0.70 µg/ml) were the only susceptible isolates to Cefpodoxime 200 mg (Ximeprox). Generally, with respect to each brand of third generation cephalosporin, isolates with mean lower MIC values were more susceptible than isolates that exhibited higher mean MICs.

However, the differences in Mean MIC observed across various groups were not statistically significant (p =  > 0.05); hence the growth of each isolate is inhibited by a specific MIC value with respect to a particular third generation cephalosporin. (That is to say, no specific mean MIC value is more effective in inhibiting the growth of two or more isolates with respect to a particular brand of third generation cephalosporin, implying that clinicians have a wide range of MIC values to select in order to optimize antibiotic therapy, given that the observed isolates exhibited a reduced susceptibility against brands of third generation cephalosporin as compared to the clinical breakpoints (CLSI).

## Discussion

Microbiological evaluation of wound swabs revealed an overall prevalence of 92% isolates in chronic wound infection while 8% of the chronic wound swabs exhibited no growth (Fig. 1). However, this high overall prevalence of isolates in chronic wound infection was not expected because several studies with larger sample size reported lower overall prevalence 13.1%(28/213)^17^. Swabs from chronic wound infection examined with routine culture methods are expected to have lower overall prevalence because 60% of chronic wounds have isolates encased in biofilms whose growth is poor with routine culture^18,19^. Although studying biofilms was beyond the scope of this study, this high observed overall prevalence as a result of using routine culture method implied that the collected samples had limited biofilms. Furthermore, higher prevalence of chronic wound isolates particularly in surgical ward of Mbarara Regional Referral Hospital could be as result of high incidence of wound contamination during dressing and inadequate infection prevention and control practices and high rate of emergency surgical operations that are likely to compromise the standard of operating procedures as reported from one study conducted in the same setting^20^. In addition, the high burden of isolates could be attributed to differences in surgical procedures, aseptic techniques as compared to other clinical setting^20–22^.

The most prevalent isolates found in chronic wound infections were *staphylococcus aureus (40.6%)*, *Klebsiella* spp. (29%) and *Proteus* spp. (21.7%); (Fig. 2). Although the observed prevalence of isolates in chronic wound infection is higher compared to other clinical settings, a similar pattern of prevalence has been report in other clinical settings^23,24^. *Staphylococcus aureus* is particularly high because the skin is a natural habitat for *staphylococcus species* hence a high risk chronic wound infections^25^. *Providencia* spp. (1.4%) and *Enterobacter agglomerans* (2.9%) were identified from the study participants on the surgical ward of MRRH whereas Escherichia coli and *acinobacter* are not common but widely prevalent in other clinical settings^23,26^. This observed difference in the pattern of prevalence across the various clinical settings could be as a result of divergent geographic distribution of microorganisms and regional differences in antibiotic resistance pattern as well as the level of antibiotic stewardship practices in clinical settings^27,28^.

Chronic wound infections caused by *staphylococcus aureus* were more prevalent among female patients (53.6%) aged below 40 years (67.9%) while chronic wound infections by *Klebsiella* spp. were most prevalent among male patients (60%) aged above 40 years (70%); (Table 3).*Enterobacter agglomerans* exhibited a similar pattern of distribution by age (50% in each age category) and gender of study participants (50% in each gender category) while *Proteus species* and *Pseudomonas aeroginosa* were more prevalent in male study participants (53.3% and 66.7% respectively) aged 40 years and below (53.3% and 66.7% respectively). All *Providentia species* chronic wound infections were only prevalent in female study participants (100%) aged 40 years and below (100%). However, statistical analysis revealed that the prevalence of chronic wound isolates was not affected by gender and age of study participants (P =  > 0.05). Similar studies in Niger delta University Teaching Hospital, a rural tertiary Hospital in Nigeria and health facilities in Cameroon also reported that gender and age of patients had no influence on the prevalence of wound isolates^29–31^. What is generally agreed is that age of a patient may affect the ability of immune response to infection with the young and elderly patients being more prone to infections which may affect bacterial isolation rate in this age groups^32^.

Because MIC values can be used in clinical settings to measure bacterial resistance and perform therapeutic drug monitoring to either optimize antibiotic therapy in case of resistant pathogens or individualize antibiotic therapy in case of organ dysfunction, this study also determined MICs of the five third generation cephalosporins against bacterial isolates. Our findings in Table 4 showed lower MICs for *staphylococcus aureus* and *Klebsiella species* against ceftriaxone1g (Epicephin). The MIC values for ceftriaxone 1 g (Epicephin) against some isolates observed in our study were even lower than those reported in Nepal teaching Hospital^33^. Our finding agrees with a previous study about surgical site infections in surgical ward of Mbarara regional referral which reported lower bacterial resistance to ceftriaxone^21^. Lower rates of bacterial resistance to ceftriaxone could be as a result of pre-authorization of ceftriaxone prescription and regular clinical audits/ward rounds led by experienced surgeons in the surgical ward of MRRH which greatly limits indiscriminate use of antibiotics, particularly ceftriaxone. In comparison, ceftriaxone resistance has been reported to be increasing in Nepal due to high incidence of irrational prescription of ceftriaxone and limited antibiotic stewardship practices across a number of health facilities in Nepal^34,35^. This finding is useful for monitoring the effectiveness of ceftriaxone 1 g (Epicephin) in patients with *Klebsiella* spp. and *staphylococcus aureus* chronic wound infections. Since all cephalosporins exhibit a time-dependent killing of pathogens, maximum plasma concentration of ceftriaxone 1 g (Epicephin) should exceed its mean MIC of 15.77 µg/ml or 22,97 µg/ml for 50–70% of the dosing interval^36^. However, increasing MIC values against ceftriaxone were observed in *Proteus species* (31.5 µg/ml), *Enterobacter agglomerans* (47.25 µg/ml) and *Pseudomonas aeruginosa* (44.65 µg/ml) respectively. Compared to the maxima plasma concentration of ceftriaxone (Table 2), the observed MIC values were much lower, suggesting that ceftriaxone (Epicephin) 1 g is still effective in the clinical management of chronic wound infections.

Additionally, the Mean MIC values of Cefoperazone + sulbactam 2 g (Sulcef) were lower for *Klebsiella* spp. (2.91 µg/ml), staphylococcus aureus 4.27 µg/ml and *Providentia species* (4.97 µg/ml) respectively. Although lower than the maximum plasma concentration of cefoperazone, higher MIC values were exhibited by *Enterobacter agglomerans* (11.19 µg/ml), *Proteus species* (8.6 µg/ml) and *Pseudomonas species* (8.5 µg/ml) respectively (Table 4). However MIC values of cefoperazone + sulbactam 2 g (Sulcef) reported from other studies were higher (16 µg/ml) as compared to MIC values (2.91–11.19 µg/ml) of pathogens in surgical ward of MRRH^37^, hence a lower rate of resistance in surgical ward of MRRH as compared to other regions. Generally, all isolates studied were susceptible at various MIC values against Cefoperazone + Sulbactam 2 g (Sulcef), therefore this drug can be reserved as a choice for empirical therapy in the management of chronic wound infections in the surgical ward of MRRH.

The findings further revealed that *enterobacter agglomerans* (0.72 µg/ml), *Klebsiella species* (1.6 µg/ml) and *staphylococcus aureus* (2.96 µg/ml) respectively exhibited lower MIC than *proteus species* (6.34 µg/ml) against cefotaxime 1 g (Omnatax) (Table 4). Overall, the MIC value of *enterobacter agglomerans Klebsiella species and staphylococcus aureus against* cefotaxime are lower than the maximum plasma concentration of cefotaxime1g (Omnatax) (Table 2), implying that cefotaxime is still effective in treatment of chronic wound infections.

It was also observed that *Staphylococcus aureus*, enterobacter agglomerans and *Proteus species* were associated with higher MIC values against Cefixime (0.40 µg/ and 0.64 µg/ml respectively) than *klebsiella species* (0.38 µg/ml).The observed MIC values in test isolates were also lower than maximum plasma concentration of cefixime, hence effective in treatment of chronic wound infections by *staphylococcus aureus, enterobacter agglomerans* and *proteus species* only. On the other hand, *Klebsiella species* and *enterobacter agglomerans* were the only susceptible isolates against cefpodoxime with MIC values of 0.34 µg/ml and 0.7 µg/ml respectively. Generally, lower MIC values imply low levels of antibiotic resistance and dosing of antibiotic therapy with lower MIC values together with pharmacokinetic/pharmacodynamics parameters of the patient increases therapeutic success^38^. Although, lower MIC values are predicted to improve the efficacy of antibiotic therapy, one study reported that sometimes lower MIC values may result in poor treatment outcome due to tolerance or persistence of pathogens in the sites of infection and heterogenous antibiotic resistance where small subpopulation continue to grow in the presence of antibiotic agent^39^.

In comparison with previous studies, the MIC values from previous studies are much lower (0.06–0.25 µg/ml) than MIC values obtained from this study with respect to Cefixime 400 mg (Gramocef-0-400) and cefpodoxime 200 mg (Ximeprox)^40^. Consequently, the continued use of cefixime (Gramocef-0-400) and cefpodoxime 200 mg (Ximeprox) in the surgical word of MRRH is likely to result into poor treatment outcomes such as longer length of hospital stay, high treatment costs, delayed wound healing and physical disability among patients with chronic wound infections^41,42^. The possible reason for higher MIC of the studied isolates against cefixime in particular is unlimited access to antibiotics through commercial pharmaceutical outlets in which it has been reported that over 40% of the antibiotics including cefixime are dispensed over-the-counter without regard of appropriate assessment of clinical indication especially in the commercial pharmaceutical outlets in Uganda^43^. Such practices of indiscriminate use of antibiotics have promoted the emergency of resistant bacterial strains hence complicating treatment of bacterial infections^44^.

We analyzed the mean MIC values of the susceptible isolates against each of the five third generation cephalosporins using one-way ANOVA to determine the mean difference in MICs. Although the results of this study did not find statistically significant differences in mean MICs, all isolates identified exhibited very high MIC values compared to the clinical breakpoints published by CLSI 2018, indicating that there is reduction in susceptibility to third generation cephalosporins and high doses may be required to achieve adequate treatment outcomes^45^.

### Study strength

This study was conducted in Mbarara University of science and technology Microbiology laboratory accredited by the central public health laboratories of Uganda (CPHL) and that follows standard and quality procedures to ensure quality and reproducible laboratory findings.

## Study limitations

- Pathogens encased in biofilms were not studied due to lack of standard microbiological methods for identification of microorganisms in biofilms. Therefore, the study results regarding prevalence of pathogens in chronic wound infection were affected.
- Patients who were currently on antibiotic therapy were not excluded from this study because part of this research study assessed “exposure to third generation cephalosporins” as one of the risk factors for antibiotic resistance. This could have affected bacterial growth.
- The sample size of study participants was small which limits generalizability of our findings.
- There was no similar study of chronic wound infection in Uganda and Africa, therefore the prevalence rate used during sample size calculation affected the sample size for this study.

### Recommendations

1. The clinicians and laboratory personnel should periodically monitor the prevalence and susceptibility pattern of pathogens that commonly infect chronic wounds against third generation cephalosporins.
2. Cefoperazone + sulbactam 2 g (Sulcef) should be prescribed as empirical therapy in the management of chronic would infection because all pathogens (6/6) commonly infecting chronic wounds in the surgical ward of MRRH were susceptible to this brand of third generation cephalosporin.
3. Clinicians in the surgical ward of MRRH need to use the observed MIC values to optimize the dosing of third generation cephalosporin because isolates had higher MIC values compared to the CLSI 2018 clinical breakpoints, indicating a reduced level of susceptibility.
4. Further studies should be carried out to determine the minimum bactericidal concentrations of the third generation cephalosporins investigated in this study.

## Conclusion

The overall prevalence of isolates in chronic wound infection among patients admitted in the surgical ward of MRRH was 92% and the most prevalent isolates were *Staphylococcus aureus, Klebsiella species* and *proteus species* respectively. The age and gender of study participants had no significant influence on the observed prevalence of isolates in chronic wound infection.

The observed MIC values were higher than the CLSI 2018 clinical breakpoint, implying a decreasing trend in susceptibility of chronic wound isolates to third generation cephalosporins at the surgical ward of MRRH.

## Supplementary Information

Supplementary Data.

## Data Availability

The data is available as supplementary information with this article.
